# Supportive Resources, Work Engagement, and Contextual Performance Among Nurses: A Cross-Sectional Mediation Study

**DOI:** 10.3390/healthcare14193254

**Published:** 2026-10-01

**Authors:** Hamza Moafa, Ahmed Nahari, Monir Almotairy, Eman Alarood

**Affiliations:** 1 Community and Psychiatric Mental Health Nursing Department, College of Nursing, King Saud University, P.O. Box 642, Riyadh 11421, Saudi Arabia; 2 Department of Family and Community Health Nursing, Dr. Sulaiman Al Habib College for Knowledge, Riyadh 13325, Saudi Arabia; eman.alarood@drsulaimanalhabib.edu.sa or 447920768@student.ksu.edu.sa; 3 Medical Surgical Nursing Department, College of Nursing, King Saud University, P.O. Box 642, Riyadh 11421, Saudi Arabia; anahari@ksu.edu.sa or ahmed.nahari@drsulaimanalhabib.edu.sa; 4 Department of Adult Health Nursing, Dr. Sulaiman Al Habib College for Knowledge, Riyadh 13325, Saudi Arabia; 5 Nursing Administration and Education Department, College of Nursing, King Saud University, P.O. Box 642, Riyadh 11421, Saudi Arabia; malmotairy@ksu.edu.sa or monir.almotairy@drsulaimanalhabib.edu.sa; 6 Department of Health Systems and Nursing Education, Dr. Sulaiman Al Habib College for Knowledge, Riyadh 13325, Saudi Arabia; 7 Master of Science in Nursing Program, College of Nursing, King Saud University, P.O. Box 642, Riyadh 11525, Saudi Arabia

**Keywords:** Saudi Arabia, work engagement, job resources, contextual performance

## Abstract

**Highlights:**

**What are the main findings?**

- Among nurses in Saudi Arabia, perceived supportive resources were positively associated with both work engagement and contextual performance.
- Work engagement statistically accounted for a modest part of the association between perceived supportive resources and contextual performance.

**What are the implications of the main findings?**

- These findings suggest that healthcare organizations may benefit from the building of supportive environments and strengthening of nurses’ work, personal, and interpersonal resources, all of which were associated with higher engagement and contextual performance in this study, with work resources showing the strongest independent association with contextual performance.
- Supportive resources appear to be associated with contextual performance partly through work engagement; nurses reporting higher work engagement also reported higher contextual performance, although these cross-sectional data cannot establish that changing engagement would change performance.

**Abstract:**

**Background:** Nurses’ contextual performance, defined as voluntary, extra-role behaviors that support colleagues, teams, and the organization, is critical to patient safety and care quality. Although individual, work, and interpersonal resources have each been linked to nurse outcomes, the pathways through which these resources are associated with contextual performance remain insufficiently examined in the nursing workforce. This study examined the relationships between individual, work, and interpersonal resources; work engagement; and contextual performance among nurses. **Methods:** A cross-sectional correlational study guided by the Job Demands–Resources model was conducted among 136 nurses working in healthcare settings in Saudi Arabia between 5 February and 11 March 2026. Data were collected using the Thriving in Nursing Questionnaire, the Utrecht Work Engagement Scale, and the Individual Work Performance Questionnaire. Descriptive statistics, Pearson correlations, multiple linear regression, and bootstrapped mediation analyses (PROCESS Model 4, 5000 resamples) were performed. **Results:** The sample was predominantly female (70.6%) and non-Saudi (63.2%), and most held a bachelor’s degree (75.0%), with a mean age of 32.73 years (SD = 6.92). Individual, work, and interpersonal resources and work engagement were positively correlated with contextual performance (*p* < 0.001). In the regression model, work resources (β = 0.40, *p* = 0.004), work engagement (β = 0.29, *p* < 0.001), and nationality (β = −0.21, *p* = 0.025; lower scores among non-Saudi nurses) were independent predictors, together explaining 35.3% of the variance (adjusted R^2^ = 0.29). Bootstrapped indirect effects of individual, work, and interpersonal resources on contextual performance through work engagement were statistically significant, indicating that work engagement statistically accounted for a modest part of each resource–performance association, with significant direct associations remaining. **Conclusions:** In the mediation models, individual, work, and interpersonal resources were associated with nurses’ contextual performance both directly and indirectly through work engagement. All variables were measured at a single time point, so these indirect effects represent statistical associations rather than causal mechanisms. These findings suggest that organizational strategies strengthening nurses’ work, personal, and interpersonal resources, particularly work resources, may support engagement and contextual performance.

## 1. Introduction

In healthcare settings, nurses play a central role in the delivery of high-quality patient care and in improving health outcomes. In addition to carrying out their assigned clinical duties, nurses are expected to demonstrate contextual performance: voluntary, extra-role behaviors such as helping colleagues, cooperating with the team, adaptability, and supporting the work environment [1,2]. These behaviors are especially important in complex and dynamic healthcare systems, where rising service demands and workforce challenges require sustained coordination and responsiveness. Research has indicated that nurses’ work-related behaviors are closely associated with patient safety, quality of care, and organizational outcomes. For instance, burnout has been associated with poorer patient safety and lower quality of care [3], while higher levels of work engagement have been linked to better patient outcomes and stronger safety indicators [4]. These findings point to the importance of understanding the factors that may enhance nurses’ performance beyond task execution.

Nurses’ performance is shaped not only by clinical competence but also by the resources available to them within their work environment. These resources can be organized across several domains. Work-related resources, such as autonomy, adequate staffing, and supportive working conditions, have been consistently associated with higher levels of performance and job satisfaction [5,6]. At the individual level, personal resources such as resilience, self-efficacy, and professional competence enable nurses to cope effectively with workplace demands and maintain positive functioning under pressure. Evidence from clinical settings, including studies conducted in Saudi Arabia, has indicated that both personal and job-related factors are central to nurses’ work engagement and performance [7,8]. Beyond these domains, interpersonal resources such as emotional support, effective leadership, and positive team relationships have been identified as contributors to engagement and care quality [9,10]. These findings suggest that different forms of resources may jointly relate to nurses’ engagement and performance.

Work engagement has emerged as a key motivational state that may help explain how resources relate to performance. Work engagement is characterized by vigor, dedication, and absorption. It reflects a positive and fulfilling work-related state that may enable nurses to invest energy and commitment in their roles. Research has shown that higher levels of engagement were associated with stronger organizational commitment and better performance outcomes in nursing practice [11]. Recent studies have suggested that engagement may serve as an underlying mechanism linking various resources to voluntary work behaviors. For example, psychological and work-related resources have been shown to enhance organizational citizenship behaviors through increased engagement among nurses [12].

Although contextual performance is recognized as a key dimension of nursing job performance, research to date has not sufficiently examined the factors and mechanisms that shape it. Only a few studies have measured contextual performance as a separate outcome. Aboagye et al. found that safety behavior mediated the link between risk perception and contextual performance [13], while Ma et al. showed that psychological capital and organizational identification mediated the effect of organizational support on performance, including its contextual dimension [14]. However, these represent isolated efforts rather than a consistent body of evidence. Other studies either treated contextual performance as a mediator rather than an outcome [15] or examined it outside the nursing profession [16]. Several factors have been shown to influence nurses’ overall performance, such as emotional intelligence [1], work motivation [17], sleep quality [18], occupational calling [19], presenteeism [20], and professional identity [21]; however, their joint role in shaping contextual performance specifically remains unclear.

The interplay among individual, work, and interpersonal resources and their associations with contextual performance through engagement remains insufficiently examined in nursing contexts. This is particularly relevant to Saudi Arabia, where regional evidence is limited and ongoing healthcare transformations highlight the need to examine how these resources relate to contextual performance. The present study therefore had two complementary aims: first, to examine the relationships among individual, work, and interpersonal resources; work engagement; and contextual performance among nurses working in healthcare settings in Saudi Arabia; and second, to test whether work engagement statistically accounts for these relationships, consistent with its proposed mediating role in the Job Demands–Resources model.

**H1.**  *Individual, work, and interpersonal resources are positively associated with contextual performance.*

**H2.**  *Work engagement mediates the association between these resources and contextual performance.*

### Theoretical Framework

**Figure 1:**
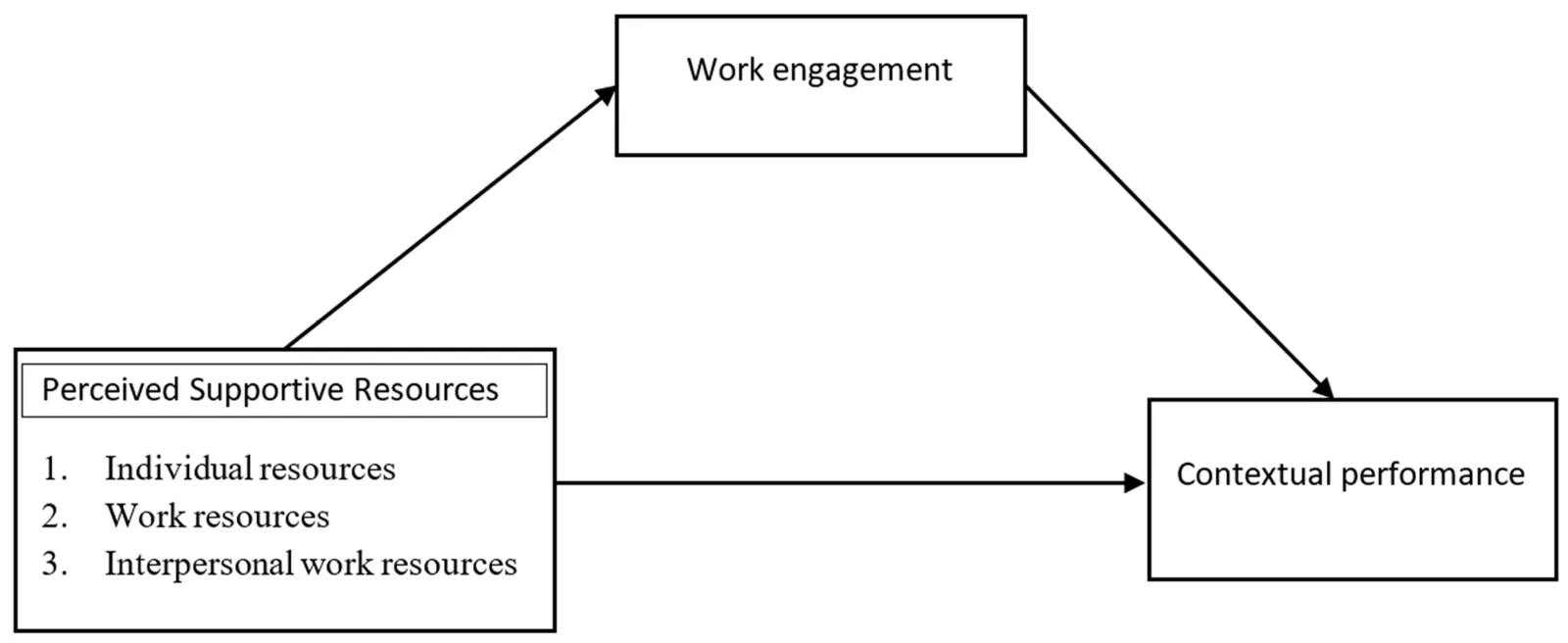
The study framework showing the hypothesized indirect association through work engagement.

This study was guided by the Job Demands–Resources (JD-R) model [22,23], which explains how work characteristics influence employee engagement and performance, and was applied with reference to recent empirical work in which engagement mediates resource–performance links [24], as illustrated in Figure 1. The JD-R model specifies the relationships among resources, work engagement, and work outcomes, regardless of occupation or hierarchical level. According to the JD-R motivational process, the availability of resources activates positive psychological states such as energy and full immersion at work that produce elevated levels of work engagement [22,23], and engagement, in turn, is linked to performance behavior [24]. Within this framework, resources serve as the motivational antecedent, work engagement as the psychological mediator, and contextual performance as the distal outcome.

## 2. Materials and Methods

### 2.1. Study Design

This study used a descriptive, cross-sectional correlational design.

### 2.2. Participants and Setting

The study included nurses working in healthcare settings in Saudi Arabia. Data were collected between 5 February and 11 March 2026, using an online survey administered through Qualtrics (Provo, UT, USA). A non-probability sampling approach combining purposive, convenience, and snowball recruitment techniques was used. Eligible participants were invited to share the survey link with colleagues meeting the inclusion criteria. Invitations containing the survey link were distributed online via the professional and personal networks of nurses. Region of employment was not captured by the demographic questionnaire; therefore, the geographical distribution of respondents could not be reported. These data were drawn from a larger research project. One previous study using data from this project has been published [25]. Of the 136 nurses analyzed here, 128 (94.1%) were also included in that study, and both studies used the same demographic items and THINQ resource subscales. The two studies are nevertheless distinct: the earlier study examined thriving at work as the mediator between supportive resources and counterproductive work behaviors, whereas the present study examines work engagement (UWES-3) as the mediator between supportive resources and contextual performance (IWPQ). These engagement and performance data, as well as all analyses reported here, have not been published previously.

The inclusion criteria were: (1) registered nurses currently employed in clinical settings in Saudi Arabia; (2) a minimum of one year of clinical experience; and (3) the ability to complete the questionnaire in English. Based on a priori power analysis conducted using G*Power (version 3.1; Heinrich-Heine-Universität Düsseldorf, Düsseldorf, Germany), the estimated sample size was 134 participants to achieve a statistical power of at least 80%, assuming a type I error rate of 0.05, a medium effect size (f^2^ = 0.15), and an anticipated 5% rate of missing data. The test specified was an F test for linear multiple regression (fixed model, R^2^ deviation from zero) with 12 predictors, corresponding to the regression model reported in the Results. The indirect effects were not separately powered. A total of 190 responses were recorded. Fifty-three respondents gave consent but did not finish the instruments analyzed in the present study and were therefore excluded, leaving 137 complete cases. Analyses were conducted on complete cases; one additional case was subsequently excluded as an influential observation (see Section 2.4), yielding a final analytical sample of 136.

### 2.3. Measurements

The study survey included demographic items and three self-reported scales. Demographic and work-related variables included age, gender, educational level, years of experience, nationality, nurse role, department/unit, practice setting, and work shift.

#### 2.3.1. Supportive Resources

Individual, work, and interpersonal resources were measured using the Thriving in Nursing Questionnaire (THINQ), a 15-item self-report instrument psychometrically validated for nurses [26]. It comprises three subscales: individual resources (three items; rated 0 = very poor to 10 = very good), work resources (six items), and interpersonal work resources (six items; both rated 0 = completely disagree to 10 = completely agree). Each subscale is scored separately by summing its items, with higher scores reflecting stronger perceived resources in that domain. No composite total is computed, as the three subscales represent conceptually distinct resource domains [26]. Individual resources capture personal strengths, concentration, and overall health; work resources reflect structural and professional work features (e.g., staffing adequacy and skill development); and interpersonal resources capture relational and organizational factors (e.g., supervisor support and unit decision-making involvement). Original-study reliabilities were McDonald’s ω = 0.87, 0.83, and 0.88 [26]. In the present sample, Cronbach’s α was 0.88 for individual and work resources and 0.85 for interpersonal resources.

#### 2.3.2. Work Engagement

Work engagement was measured using the Utrecht Work Engagement Scale (UWES), which conceptualizes engagement as a positive, work-related state of mind characterized by vigor, dedication, and absorption [27]. The ultra-short version (UWES-3) was selected to minimize respondent burden while retaining the core construct, and because the present study modeled engagement as a global mediator rather than examining its subdimensions separately [28]. The UWES-3 correlates strongly with the original nine-item version (UWES-9) and has demonstrated satisfactory reliability across national samples, supporting its validity as a global measure of work engagement [28]. The three items are rated on a 7-point scale ranging from 0 (never) to 6 (always), and the total score is calculated as the mean of the three items, with higher scores indicating greater work engagement [28]. Cross-national validation across five countries reported reliability coefficients ranging from 0.77 to 0.85, supporting its use as a valid alternative to the longer UWES versions [28]. In the current study, the UWES-3 demonstrated acceptable internal consistency (Cronbach’s α = 0.73).

#### 2.3.3. Contextual Performance

Contextual performance was measured using the Individual Work Performance Questionnaire (IWPQ), which conceptualizes contextual performance as behavior supporting the organizational, social, and psychological environment in which technical work is conducted, beyond the core technical requirements of the job [29]. The contextual performance subscale was used to capture this domain of job performance, consistent with the present study’s focus on extra-role behaviors that sustain the broader work environment rather than task-specific output [29]. The IWPQ subscales have demonstrated satisfactory reliability and construct validity across occupational samples, supporting their use as valid measures of individual work performance [29]. The eight items are rated on a 5-point scale ranging from 0 (rarely) to 4 (always), and the total score is calculated as the mean of the eight items, with higher scores indicating greater contextual performance [29]. The original validation study reported acceptable internal consistency for the contextual performance subscale (α = 0.85), supporting its use as a reliable measure of this performance domain [29]. In the current study, the contextual performance subscale demonstrated excellent internal consistency (Cronbach’s α = 0.88).

### 2.4. Data Analysis

Descriptive statistics summarized participants’ characteristics and study variables. Sociodemographic variables (gender, nurse role, qualification, shifts, healthcare facility, and unit intensity) were dichotomized to ensure adequate cell sizes for regression modeling and to avoid unstable estimates arising from sparse categories. Pearson’s (r) and point-biserial correlations examined associations among age, experience, individual, work, and interpersonal resources, work engagement, contextual performance, and dichotomous sociodemographic variables. Regression assumptions were checked, and one influential case, identified by a standardized residual exceeding ±3 (3.09) and a Cook’s distance above the conventional 4/N cut-off (D = 0.098 vs. 4/137 = 0.029), was removed, yielding a final sample of 136. Although age and nursing experience were correlated, both were retained as theoretically relevant covariates, as their variance inflation factors remained below 10, indicating no problematic multicollinearity. An independent-samples *t*-test confirmed no significant differences between frontline and non-frontline nurses on study variables, supporting their treatment as one group. Mediation analysis was performed using PROCESS Model 4 with 5000 bootstrap samples [30]. Each resource subscale was tested in a separate model to estimate its indirect effect through work engagement, consistent with the THINQ scoring structure, in which the three subscales are independently scored without a composite total [26]. No covariates were entered in the mediation models, as covariate adjustment was not part of the mediation research question; these unadjusted estimates were therefore interpreted separately from the covariate-adjusted regression model. Given the single-time-point data, indirect effects are reported as statistical associations and are not taken to demonstrate causal mediation or temporal ordering. Significance was set at *p* < 0.05. Analyses used SPSS version 31 (IBM Corp., Armonk, NY, USA).

### 2.5. Ethical Considerations

This study received institutional review board approval from King Saud University (Ref. No. KSU-HE-25-1401). An electronic information sheet was provided as part of the online survey, outlining the study’s objectives, eligibility, voluntary participation, confidentiality, and the right to withdraw without consequence. All responses were anonymized, coded, and securely stored. Participants provided informed consent electronically before accessing the survey items; completion of the survey was conditional on this consent.

## 3. Results

A total of 136 nurses were included in the final analysis. The mean age was 32.73 years (SD = 6.92), with an average of 9.15 years of professional experience (SD = 6.47). The sample was predominantly female (70.6%) and non-Saudi (63.2%), and most held a bachelor’s degree in nursing (75.0%). The majority were frontline nurses (70.6%) working rotating shifts (54.4%). Sample characteristics and descriptive statistics of the study variables are presented in Table 1.

### 3.1. Correlation Analysis

Pearson correlation analysis revealed that all resources and performance variables were significantly and positively intercorrelated (all *p* ≤ 0.004). As shown in Table 2, contextual performance was most strongly correlated with work resources (r = 0.446, *p* < 0.001), followed by interpersonal work resources (r = 0.378, *p* < 0.001) and individual resources (r = 0.376, *p* < 0.001). A moderate positive correlation was also observed between contextual performance and work engagement (r = 0.395, *p* < 0.001). Work engagement was significantly correlated with interpersonal work resources (r = 0.302, *p* < 0.001), individual resources (r = 0.291, *p* < 0.001), and work resources (r = 0.246, *p* = 0.004). Among the three resource dimensions, interpersonal work resources exhibited the strongest association with work engagement, while work resources showed the weakest. The correlation findings provide preliminary support for the hypothesized relationships among job resources, work engagement, and contextual performance, warranting further examination through regression and mediation analysis. None of the demographic or work-related variables were significantly correlated with contextual performance or work engagement (Table 2).

### 3.2. Regression Analysis

As shown in Table 3, a multiple linear regression was conducted to examine the extent to which individual resources, work resources, interpersonal work resources, and work engagement, together with demographic and work-related characteristics (age, gender, qualification, nursing experience, nationality, unit intensity, shift type, and nurse role), predicted contextual performance among nurses. The overall model was statistically significant, F(12, 123) = 5.59, *p* < 0.001, and accounted for 35.3% of the variance in contextual performance (R^2^ = 0.35, adjusted R^2^ = 0.29; Cohen’s f^2^ = 0.55). The Durbin–Watson statistic (2.19) indicated no autocorrelation of residuals, and tolerance values were above 0.14, with variance inflation factors below 10, suggesting that multicollinearity was not a concern, although age and nursing experience showed elevated VIFs (6.79 and 6.84, respectively), reflecting their conceptual overlap.

Three predictors were independently associated with contextual performance. Work resources emerged as the strongest predictor, B = 0.04, SE = 0.01, β = 0.40, t(123) = 2.96, *p* = 0.004, 95% CI [0.01, 0.06], indicating that higher levels of perceived work resources were associated with higher contextual performance. Work engagement was also a significant positive predictor, B = 0.25, SE = 0.07, β = 0.29, t(123) = 3.73, *p* < 0.001, 95% CI [0.12, 0.38]. Nationality was negatively associated with contextual performance, B = −0.39, SE = 0.17, β = −0.21, t(123) = −2.27, *p* = 0.025, 95% CI [−0.73, −0.05], indicating that non-Saudi nurses reported lower contextual performance than Saudi nurses, after adjusting for the other variables in the model. The remaining predictors were not significantly associated with contextual performance.

### 3.3. Mediation Analysis

**Figure 2:**
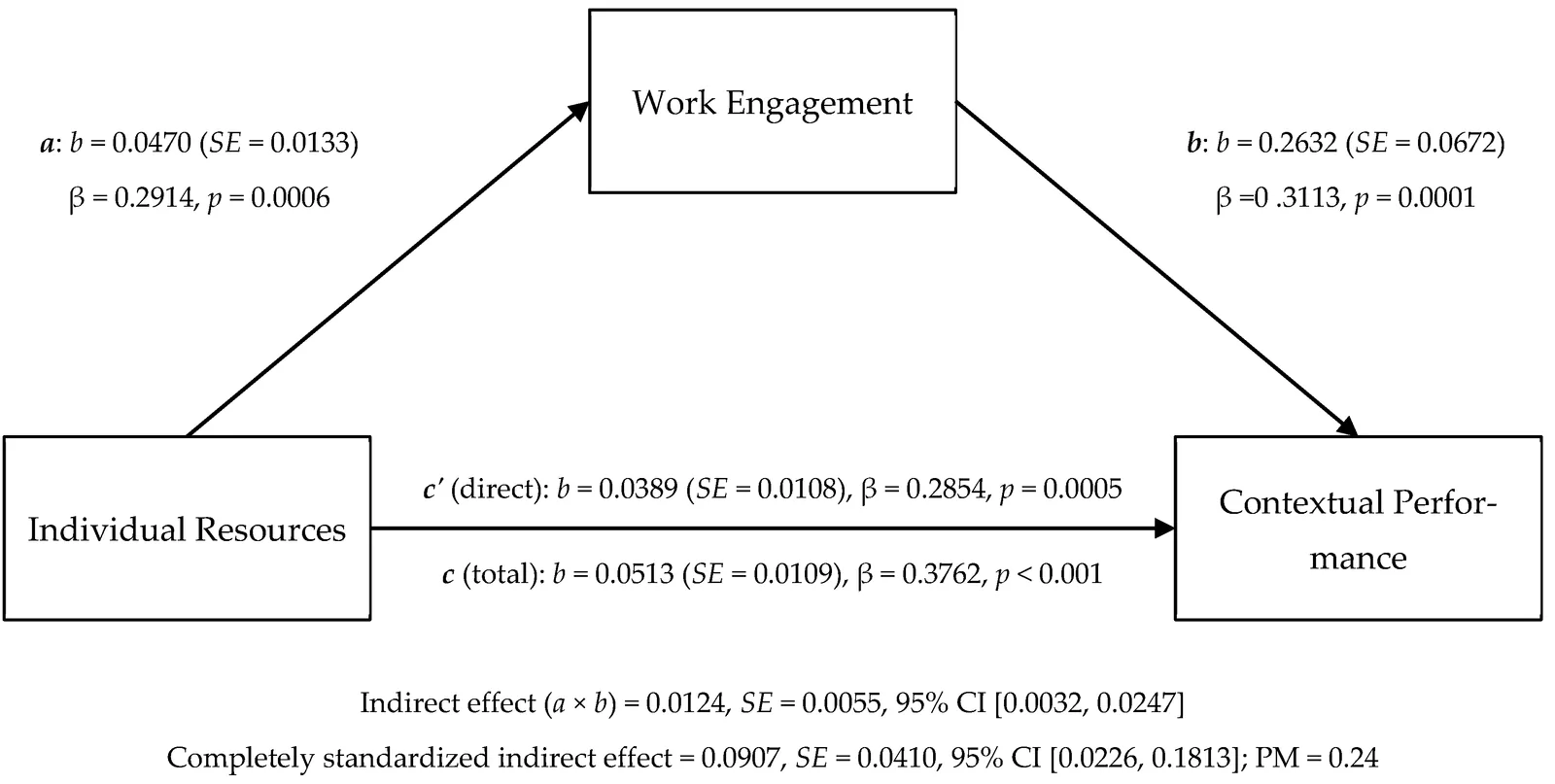
Indirect-association model linking individual resources to contextual performance through work engagement.

**Figure 3:**
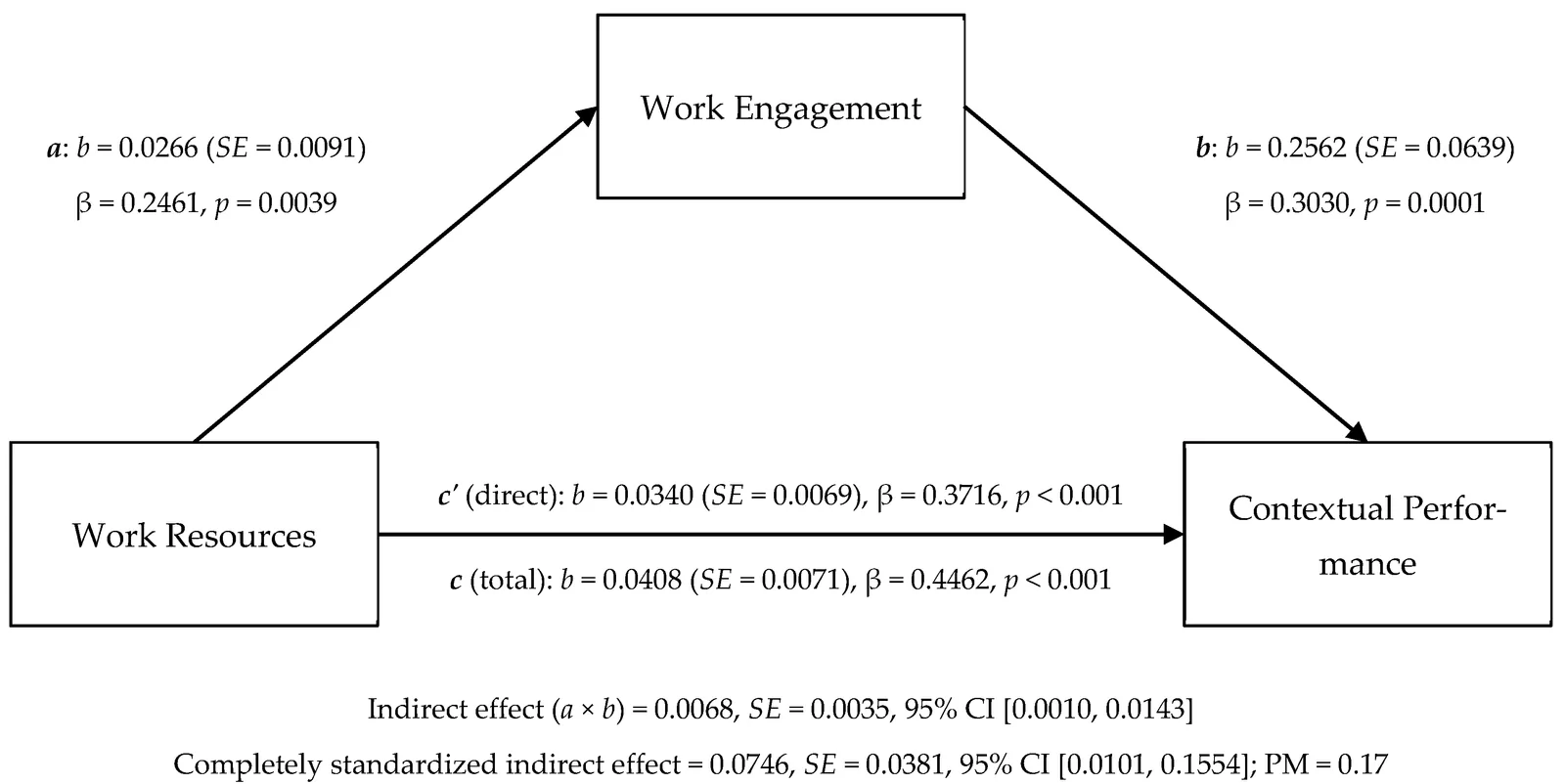
Indirect-association model linking work resources to contextual performance through work engagement.

**Figure 4:**
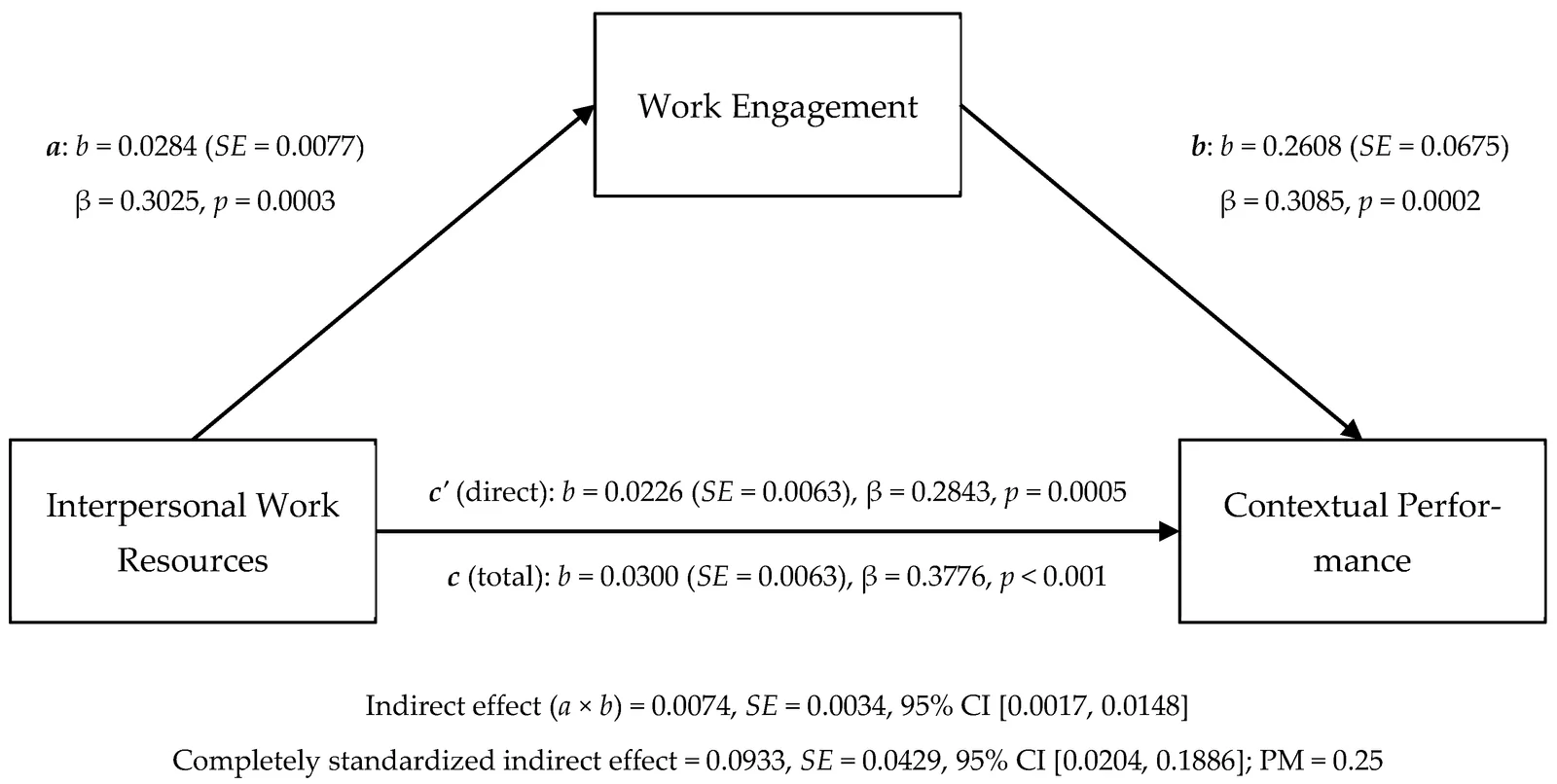
Indirect-association model linking interpersonal work resources to contextual performance through work engagement.

A consistent pattern of results emerged across all three models (Table 4; Figure 2, Figure 3 and Figure 4). Each resource dimension was positively associated with work engagement (path a): individual resources (β = 0.2914, *p* = 0.0006; see Figure 2), work resources (β = 0.2461, *p* = 0.0039; see Figure 3), and interpersonal work resources (β = 0.3025, *p* = 0.0003; see Figure 4). Work engagement, in turn, was positively associated with contextual performance after controlling for the respective predictor in each model (path b; βs = 0.3030–0.3113, all *p* values ≤ 0.0002). The total associations between each predictor and contextual performance were statistically significant (path c; βs = 0.3762–0.4462, all *p* values < 0.001). After work engagement was included in the models, these associations were attenuated but remained significant (path c′; βs = 0.2843–0.3716, all *p* values ≤ 0.0005), indicating partial rather than full mediation. The percentile bootstrap confidence intervals for all three indirect effects excluded zero: individual resources (b = 0.0124, SE = 0.0055, 95% CI [0.0032, 0.0247]; completely standardized = 0.0907, SE = 0.0410, 95% CI [0.0226, 0.1813]; Figure 2), work resources (b = 0.0068, SE = 0.0035, 95% CI [0.0010, 0.0143]; completely standardized = 0.0746, SE = 0.0381, 95% CI [0.0101, 0.1554]; Figure 3), and interpersonal work resources (b = 0.0074, SE = 0.0034, 95% CI [0.0017, 0.0148]; completely standardized = 0.0933, SE = 0.0429, 95% CI [0.0204, 0.1886]; Figure 4). These findings collectively indicate that work engagement statistically accounted for a modest part of the observed associations between each resource dimension and contextual performance.

## 4. Discussion

This study examined the relationships among individual, work, and interpersonal resources; work engagement; and contextual performance among nurses in Saudi Arabia. All three resource dimensions and work engagement were positively related to contextual performance. In the adjusted regression model, work resources, work engagement, and nationality remained significant predictors, and work engagement statistically accounted for a modest part of the association between each resource dimension and contextual performance.

Compared with previous studies, the level of work engagement in the present sample was lower than that reported among Saudi hospital nurses [7]. Levels of individual, work, and interpersonal resources were comparable to those reported among nurses in the United States [31]. The level of contextual performance was comparable to that reported for staff nurses in Saudi Arabian hospitals, but lower than that reported for nurse managers in the same study [5]. These differences may reflect variations in work settings, roles, and workforce composition across samples, as well as differences in how the constructs were measured.

A central finding is the positive association between work engagement and contextual performance: more engaged nurses reported more helping, organizational support, and climate-maintaining behaviors, consistent with prior evidence that engaged nurses perform more strongly [4,32]. Engagement has also been linked to patient outcomes. A systematic review and meta-analysis of healthcare staff found a small but consistent association between staff engagement and patient safety, including fewer self-reported errors and adverse events [33]; unit-level job engagement was associated with fewer errors, higher patient safety, and higher patient satisfaction [11]; a meta-analysis of over 288,000 nurses linked burnout, the opposite of engagement, to more medication errors, adverse events, and lower quality of care [3]; and a national U.S. survey found that more engaged nurses reported better perceived care quality and lower intent to leave [4]. Engagement therefore appears relevant not only to retention but also to clinical performance and patient safety.

Individual resources were positively associated with both engagement and contextual performance. This accords with evidence that psychological capital, including self-efficacy, optimism, resilience, and hope, predicts engagement and collaborative behavior, even under adversity [34]; that resilience and self-efficacy relate to engagement, with self-efficacy mediating the relationship between resilience, stress, and engagement [8]; that psychological capital relates to organizational citizenship behavior through engagement [12]; and that emotional intelligence, resilience, and psychological empowerment are associated with engagement and extra-role behavior among critical care nurses [35]. In the adjusted regression model, however, individual resources were no longer a significant predictor once engagement and the other resources were controlled. As the mediation models were unadjusted, the two sets of results address different questions and should not be treated as interchangeable. In the mediation model, individual resources were associated with engagement, which in turn was associated with contextual performance, with a significant indirect effect and a significant remaining direct association, consistent with partial mediation. Nurses’ personal strengths may therefore relate to performance partly, but not mainly, through their association with engagement.

Work resources were positively associated with both engagement and contextual performance, consistent with the JD-R proposition that job resources promote motivation and engagement, leading to enhanced performance [22]. An integrative review of reviews identified supervisor support, fair and authentic management, transformational leadership, interpersonal relations, autonomy, and professional resources as the six key job resources linked to motivational and health-related outcomes among nurses [6], and a systematic review of 61 studies reported that organizational support predicted engagement and that optimism mediated the relationship between autonomy and organizational citizenship behavior, illustrating how work and personal resources may jointly relate to contextual performance [36]. Work resources remained a significant predictor in the adjusted regression model, and in the unadjusted mediation model their direct association with contextual performance also remained significant after engagement was included, consistent with partial mediation. Thus, supportive environments may be linked both to engagement and to the practical conditions for contextual behavior. Adequate staffing, effective communication, leadership support, and participative decision-making may therefore support both engagement and performance, although this requires confirmation in intervention studies.

Interpersonal resources were likewise positively associated with both engagement and contextual performance. In a two-wave longitudinal study, emotional support from supervisors and co-workers was positively related to engagement over time, which, in turn, mediated its relationships with perceived quality of care and organizational citizenship behavior [10], suggesting that engagement is one pathway through which supportive relationships relate to contextual performance. In a large cross-sectional study of 2541 nurses across nine public hospitals in Spain, supervisor–nurse relationship quality was positively correlated with organizational citizenship behavior [9], and in China, colleague solidarity predicted both task and contextual performance, with positive emotions mediating this relationship [37]. Interpersonal resources did not, however, remain a significant predictor in the adjusted regression model, whereas the unadjusted mediation model showed both a significant indirect association through engagement and a significant direct association, consistent with partial mediation; because the models were adjusted differently, these results should not be treated as interchangeable. The loss of significance for individual and interpersonal resources in the adjusted model should not be read as evidence of no association; with a modest sample and several intercorrelated predictors, the model has limited power to separate their effects, and these coefficients are better described as imprecise than as null.

Apart from nationality, demographic characteristics were not significantly associated with contextual performance, suggesting that, in this sample, contextual performance was more closely associated with psychological and organizational factors than with age, gender, or experience. The lower contextual performance reported by non-Saudi nurses warrants careful interpretation. Nationality was unrelated to contextual performance at the bivariate level and reached significance only after adjustment for the other predictors, so the finding should be treated as tentative and may reflect suppression or confounding rather than a substantive group difference. The study did not measure factors that might account for such a difference, which merits investigation in samples designed for that purpose; prior research on demographic influences on nurse performance has been mixed [38].

The partial mediation observed for all three resource types is consistent with the JD-R framework, in which personal and job resources stimulate engagement and thereby performance [22], and adds to JD-R research by identifying engagement as a statistical pathway linking resources to contextual performance among nurses. The three resource domains showed comparable indirect associations, with individual and interpersonal work resources slightly stronger than work resources, suggesting that psychological, relational, and structural resources are each associated with engagement rather than any single domain being dominant. Engagement nevertheless accounted for only a modest share of each resource–performance association, with most of the association remaining direct (Table 4), so it appears to be one of several routes rather than the principal one. Its share was smallest for work resources, suggesting that structural resources may support contextual behaviors directly, for example, by providing the time and staffing that allow nurses to assist colleagues. Given the cross-sectional design and the small differences between domains, these patterns should be regarded as hypotheses for future testing rather than as a ranking. In practical terms, engagement-focused programs alone may be insufficient, and staffing, communication, and nurses’ involvement in decision-making warrant attention in their own right.

Beyond these general implications, the findings should also be interpreted in light of the Saudi healthcare context. Nearly two-thirds of the nurses in this sample were non-Saudi, which suggests that much of the contextual performance reported here, such as helping colleagues and supporting team functioning, likely occurred within culturally diverse teams. Whether workforce composition or cultural factors modify the associations observed in this study could not be examined with the present data and should be addressed in future research.

Given that work resources and engagement were the strongest predictors of contextual performance, healthcare administrators may wish to prioritize strengthening of the work environment and support for engagement. Although the overall model explained a substantial share of the variance in contextual performance, engagement accounted for only a minority of each resource–performance association, so its practical importance should not be overstated. Nurse leaders should ensure adequate staffing, encourage open communication, grant autonomy, recognize achievements, and involve nurses in decision-making. Supportive leadership should be promoted and abusive supervision avoided, as abusive supervision reduces nurses’ self-efficacy and engagement [39], whereas transformational leadership strengthens engagement and extra-role performance through strengthened self-efficacy [40].

Training that strengthens individual resources such as self-efficacy, resilience, optimism, and psychological capital may be worth evaluating as a means of supporting engagement and performance, and mentorship, coaching, and resilience-building initiatives could help nurses develop the confidence and coping skills needed to manage high job demands [41]. Organization-directed interventions that promote team climate and social support within nursing units, which have been reviewed for their effects on healthcare professionals’ well-being [42], may also be relevant given the indirect association of interpersonal resources with contextual performance through engagement observed here. These recommendations follow from observed associations rather than demonstrated intervention effects and require confirmation, including of their direction, in longitudinal or intervention studies.

### Limitations

The results should be interpreted with caution due to several limitations. First, because all variables were collected concurrently, the study cannot determine which variable came first or whether the associations run in the hypothesized direction; for example, nurses who perform well may, in turn, report greater engagement. The bootstrapped indirect effects are therefore descriptive estimates that fit the proposed model but do not demonstrate causal mediation. Second, purposive, convenience, and snowball sampling produced a self-selected sample recruited through informal professional networks, so the sample cannot be assumed to represent the national nursing workforce; in addition, the online, English-language format may have excluded some eligible nurses. Third, the reliance on self-reported data raises the potential for common method and social desirability biases. Safeguards were procedural only: anonymous and voluntary participation, assurance of confidentiality, and separate validated instruments with differing response formats; common method variance was not statistically assessed for the set of measures analyzed here, and a single post hoc test would not, on its own, rule out this bias. Fourth, the ultra-short engagement measure does not permit vigor, dedication, and absorption to be examined as distinct facets, and its internal consistency in this sample, although acceptable, was lower than that of the other scales. Fifth, the resource domains were modeled separately, consistent with the THINQ scoring structure, so their unique indirect effects cannot be estimated with the others held constant and should not be summed. In addition, dichotomizing several demographic and work-related variables reduced the information available and may have lowered the power to detect their associations. Finally, the sample provided limited precision for small indirect effects and for separating correlated predictors such as age and nursing experience. Future research should employ longitudinal or experimental designs, include nurses sampled more broadly across regions and settings, and incorporate objective performance measures. Moreover, further investigation is warranted to determine whether the indirect association through engagement holds across different nursing specialties and healthcare contexts. Future studies with larger samples should also test covariate-adjusted mediation models.

## 5. Conclusions

This study shows that nurses’ contextual performance is associated with work engagement and individual, work, and interpersonal work resources. Although individual, work, and interpersonal resources were all associated with contextual performance, work engagement statistically accounted for only a modest part of these associations, with direct associations remaining in our cross-sectional mediation analyses. These findings suggest that healthcare organizations may benefit from the building of supportive environments and strengthening of nurses’ work, personal, and interpersonal resources, all of which were associated with higher engagement and contextual performance in this study, with work resources showing the strongest independent association with contextual performance. With all measures collected at a single time point, these findings describe patterns of association rather than causal effects, and the value of resource- and engagement-focused strategies requires confirmation in longitudinal or intervention research.

## Figures and Tables

**Table 1:** Sample characteristics and descriptive statistics (N = 136).

	Category	Mean	SD	n	%	Min–Max
Age		32.73	6.92	--	--	—
Years of experience		9.15	6.47			—
Contextual Performance		2.39	0.90	--	--	0–4
Work Engagement		3.86	1.24			0–6
Individual Resources		20.58	6.60	--	--	0–30
Work Resources		44.95	9.84			13–60
Interpersonal Work Resources		40.26	11.34	--	--	4–60
Gender	Male			40	29.4%	
	Female			96	70.6%	
Nationality	Saudi			50	36.8%	
	Non-Saudi			86	63.2%	
Qualification	Diploma			18	13.2%	
	Bachelor’s degree			102	75.0%	
	Master’s degree			16	11.8%	
Shifts	Morning shift			57	41.9%	
	Evening shift			2	1.5%	
	Night shift			3	2.2%	
	Rotating shifts			74	54.4%	
Department/Unit	Medical-Surgical			14	10.3%	
	Emergency			21	15.4%	
	Intensive Care Unit (ICU)			24	17.6%	
	Maternity/Obstetric			9	6.6%	
	Pediatric			10	7.4%	
	Outpatient Clinic			15	11.0%	
	Other			21	15.4%	
	Rehabilitation			9	6.6%	
	Oncology			2	1.5%	
	OR			5	3.7%	
	Mental Health Nursing			2	1.5%	
	Neonatal department			1	0.7%	
	Isolation			1	0.7%	
	Hemodialysis Unit			1	0.7%	
	Dental			1	0.7%	
Healthcare facility	Primary Health Center			6	4.4%	
	General Hospital			25	18.4%	
	Specialized Hospital			38	27.9%	
	Academic hospital			23	16.9%	
	Private Hospital or Clinic			35	25.7%	
	Other			6	4.4%	
	Long-term facility			3	2.2%	
Nurse role	Frontline			96	70.6%	
	Non-frontline			40	29.4%	

**Table 2:** Pearson correlations among study variables (N = 136).

Variable	Contextual Performance	*p*	Work Engagement	*p*
Contextual performance	—		0.395 **	<0.001
Work engagement	0.395 **	<0.001	—	
Individual resources	0.376 **	<0.001	0.291 **	<0.001
Work resources	0.446 **	<0.001	0.246 **	0.004
Interpersonal work resources	0.378 **	<0.001	0.302 **	<0.001
Age	0.057	0.512	0.156	0.070
Gender	0.027	0.753	−0.061	0.477
Qualification	0.027	0.759	−0.031	0.718
Nursing experience	0.004	0.965	0.082	0.341
Nationality	−0.057	0.509	0.125	0.147
Unit intensity	0.089	0.302	0.003	0.969
Shifts	−0.001	0.988	0.016	0.857
Nurse role	0.085	0.324	0.072	0.407

Note. Age and nursing experience were measured continuously in years. Gender (1 = male, 2 = female), Nationality (1 = Saudi, 2 = non-Saudi), Nurse role (1 = frontline, 2 = non-frontline), Qualification (1 = BSN or less, 2 = graduate degree), Shifts (1 = non-rotating, 2 = rotating), and Unit Intensity (1 = high intensity: Emergency, ICU, OR, Neonatal, Isolation, Hemodialysis, and Oncology; 2 = low intensity: Medical–Surgical, Maternity/Obstetric, Pediatric, Outpatient Clinic, Rehabilitation, Mental Health Nursing, and Dental). ** *p* < 0.01.

**Table 3:** Multiple linear regression predicting contextual performance.

	B	Std. Error	Beta	t	Sig.	95.0% CI Lower Bound	95.0% CI Upper Bound	Tolerance	VIF
**(Constant)**	0.127	0.876		0.145	0.885	−1.608	1.862		
**Individual resources**	0.008	0.015	0.060	0.553	0.581	−0.021	0.038	0.441	2.270
**Work resources**	0.037	0.012	0.400	2.959	0.004 **	0.012	0.061	0.288	3.470
**Interpersonal work resources**	−0.001	0.011	−0.016	−0.117	0.907	−0.022	0.020	0.297	3.361
**Work engagement**	0.247	0.066	0.292	3.725	<0.001 **	0.116	0.379	0.854	1.171
**Age**	0.019	0.025	0.145	0.770	0.443	−0.030	0.068	0.147	6.792
**Gender**	0.029	0.159	0.015	0.184	0.855	−0.285	0.343	0.808	1.237
**Qualification**	0.212	0.225	0.076	0.943	0.347	−0.233	0.657	0.805	1.242
**Nursing experience**	−0.026	0.026	−0.187	−0.988	0.325	−0.078	0.026	0.146	6.844
**Nationality**	−0.392	0.173	−0.211	−2.274	0.025 *	−0.734	−0.051	0.610	1.639
**Unit intensity**	0.157	0.142	0.086	1.101	0.273	−0.125	0.438	0.866	1.154
**Shifts**	0.246	0.159	0.137	1.544	0.125	−0.069	0.561	0.672	1.488
**Nurse Role**	0.299	0.181	0.152	1.652	0.101	−0.059	0.658	0.621	1.611

R = 0.594, R^2^ = 0.353, adjusted R^2^ = 0.29, SE = 0.758, Durbin–Watson = 2.187, F(12, 123) = 5.594, *p* < 0.001. Note. Age and nursing experience were measured continuously in years. Gender (1 = male, 2 = female), Nationality (1 = Saudi, 2 = non-Saudi), Nurse role (1 = frontline, 2 = non-frontline), Qualification (1 = BSN or less, 2 = graduate degree), Shifts (1 = non-rotating, 2 = rotating), and Unit Intensity (1 = high intensity: Emergency, ICU, OR, Neonatal, Isolation, Hemodialysis, and Oncology; 2 = low intensity: Medical–Surgical, Maternity/Obstetric, Pediatric, Outpatient Clinic, Rehabilitation, Mental Health Nursing, and Dental). * *p* < 0.05. ** *p* < 0.01.

**Table 4:** Indirect associations between resources and contextual performance through work engagement (N = 136).

Predictor/Path	β	b	SE	t	*p*	95% CI	PM
Predictor 1: Individual Resources							
Path a: Individual Resources → Work Engagement	0.2914	0.0470	0.0133	3.5261	0.0006	0.0206–0.0733	
Path b: Work Engagement → Contextual Performance (controlling Individual Resources)	0.3113	0.2632	0.0672	3.9146	0.0001	0.1302–0.3962	
Path c: Individual Resources → Contextual Performance	0.3762	0.0513	0.0109	4.6996	<0.001	0.0297–0.0729	
Path c′: Individual Resources → Contextual Performance (controlling Work Engagement)	0.2854	0.0389	0.0108	3.5892	0.0005	0.0175–0.0604	
Indirect: Individual Resources → Work Engagement → Contextual Performance	—	0.0124	0.0055	—	—	0.0032–0.0247	
Completely standardized indirect effect: Individual Resources → Work Engagement → Contextual Performance	0.0907	—	0.0410	—	—	0.0226–0.1813	0.24
Predictor 2: Work Resources							
Path a: Work Resources → Work Engagement	0.2461	0.0266	0.0091	2.9394	0.0039	0.0087–0.0445	
Path b: Work Engagement → Contextual Performance (controlling Work Resources)	0.3030	0.2562	0.0639	4.0070	0.0001	0.1297–0.3826	
Path c: Work Resources → Contextual Performance	0.4462	0.0408	0.0071	5.7720	<0.001	0.0268–0.0548	
Path c′: Work Resources → Contextual Performance (controlling Work Engagement)	0.3716	0.0340	0.0069	4.9142	<0.001	0.0203–0.0477	
Indirect: Work Resources → Work Engagement → Contextual Performance	—	0.0068	0.0035	—	—	0.0010–0.0143	
Completely standardized indirect effect: Work Resources → Work Engagement → Contextual Performance	0.0746	—	0.0381	—	—	0.0101–0.1554	0.17
Predictor 3: Interpersonal Work Resources							
Path a: Interpersonal Work Resources → Work Engagement	0.3025	0.0284	0.0077	3.6736	0.0003	0.0131–0.0437	
Path b: Work Engagement → Contextual Performance (controlling for Interpersonal Work Resources)	0.3085	0.2608	0.0675	3.8623	0.0002	0.1272–0.3944	
Path c: Interpersonal Work Resources → Contextual Performance	0.3776	0.0300	0.0063	4.7210	<0.001	0.0174–0.0425	
Path c′: Interpersonal Work Resources → Contextual Performance (controlling Work Engagement)	0.2843	0.0226	0.0063	3.5595	0.0005	0.0100–0.0351	
Indirect: Interpersonal Work Resources → Work Engagement → Contextual Performance	—	0.0074	0.0034	—	—	0.0017–0.0148	
Completely standardized indirect effect: Interpersonal Work Resources → Work Engagement → Contextual Performance	0.0933	—	0.0429	—	—	0.0204–0.1886	0.25

Note. All models were estimated using the PROCESS macro (Model 4) [30] with 5000 bootstrap samples. 95% CI = confidence interval based on LLCI and ULCI. Bootstrap CIs that exclude zero indicate a significant indirect effect. b = unstandardized coefficient; β = completely standardized coefficient. For indirect effects, SE and 95% CI are the bootstrap standard error and percentile bootstrap confidence interval of the unstandardized or completely standardized estimate, as labeled. PM = proportion mediated, calculated as the completely standardized indirect effect divided by the standardized total effect (path c).

## Data Availability

The data presented in this study are available upon request from the corresponding author. The data are not publicly available due to privacy and ethical restrictions. A subset of this dataset was analyzed in a previously published study [25], which addressed a different research question, with a different outcome, a different mediator, and a different theoretical framework. Of the 136 participants analyzed here, 128 were also included in that study. No other publications have arisen from this dataset.

## Abbreviations

The following abbreviations are used in this manuscript:

JD-R	Job Demands–Resources
THINQ	Thriving in Nursing Questionnaire
UWES	Utrecht Work Engagement Scale
IWPQ	Individual Work Performance Questionnaire
STROBE	Strengthening the Reporting of Observational Studies in Epidemiology
IRB	Institutional Review Board
CI	Confidence interval
VIF	Variance inflation factor
